# The *C*-Terminal Phenylalanine of the Thanatin-like Peptide Rip-2 Contributes to Its Enhanced LptA-Associated Antimicrobial Activity Compared with Thanatin

**DOI:** 10.3390/ijms27188072

**Published:** 2026-09-10

**Authors:** Pavel V. Panteleev, Victoria N. Safronova, Julia S. Teplovodskaya, Ilia Y. Toropygin, Ekaterina V. Nikitinskaya, Denis A. Nikitinskiy, Ilia A. Bolosov, Alexey V. Mishin, Tatiana V. Ovchinnikova

**Affiliations:** 1 M.M. Shemyakin & Yu.A. Ovchinnikov Institute of Bioorganic Chemistry, Russian Academy of Sciences, 117997 Moscow, Russia; p.v.panteleev@gmail.com (P.V.P.); victoria.saf@ibch.ru (V.N.S.); juliyateplovodskaya@gmail.com (J.S.T.); bolosov@ibch.ru (I.A.B.); 2 Moscow Center for Advanced Studies, 123592 Moscow, Russia; mishinalexej@gmail.com; 3 Institute of Biomedical Chemistry, 119121 Moscow, Russia; 4 All-Russian Plant Quarantine Center (VNIIKR), 140150 Bykovo, Russia; nikitinskajacat@yandex.ru (E.V.N.); denpreffect@gmail.com (D.A.N.)

**Keywords:** antibiotic, antimicrobial peptide, thanatin, Gram-negative bacteria, lipopolysaccharide, LptA, drug resistance

## Abstract

The proteins of the lipopolysaccharide transport (Lpt) complex have emerged as promising targets for thanatin-like antimicrobial peptides (AMPs) active against Gram-negative bacteria. Here, we investigated the molecular features associated with the enhanced antibacterial activity of the thanatin-like peptide Rip-2 from the bean bug *Riptortus pedestris*, compared with thanatin. Microscale thermophoresis showed that Rip-2 produced a concentration-dependent response with recombinant LptA_m_-sfGFP-His_6_ that differed from that for thanatin. A phenotypic counter-screening was performed to identify genetic changes associated with Rip-2-selective reduced susceptibility. Subsequent whole-genome sequencing identified the same LptA R76C substitution as a recurrent resistance-associated change in all selected clones. This substitution was associated with only a two-fold increase in the MIC of thanatin, whereas the MIC of Rip-2 was 16-fold higher. Structure-guided modeling suggested that the guanidinium group of LptA Arg76 may contribute to Rip-2 engagement by forming predicted contacts with both the terminal carboxylate and the aromatic side chain of the peptide *C*-terminal Phe18 residue. Consistent with this model, substitutions of this phenylalanine impaired the peptide antibacterial activity, with more tolerated aromatic replacements in comparison to nonaromatic ones. These findings support an important role for the Rip-2 *C*-terminal Phe18 aromatic residue in the enhanced activity of this peptide relative to thanatin and provide a framework for the rational design of more potent thanatin-derived antibiotics.

## 1. Introduction

Antibiotic resistance of Gram-negative bacteria is a major challenge for modern anti-infective therapy [1]. Multidrug-resistant *Enterobacteriaceae*, *Pseudomonas aeruginosa*, and *Acinetobacter baumannii* are especially difficult to treat due to acquired resistance mechanisms combined with the intrinsic barrier function of the Gram-negative cell envelope. This barrier is largely defined by the outer membrane structure, whose outer leaflet is formed by lipopolysaccharide (LPS). As an essential and Gram-negative-specific structure, the LPS-containing outer membrane, together with the pathways responsible for synthesizing and transporting LPS to the cell surface, provides attractive antibiotic targets that are absent in host cells [2].

LPS is synthesized at the cytoplasmic side of the inner membrane and transported to the bacterial cell surface. This process is mediated by the LPS transport complex (Lpt complex), whose canonical core consists of seven essential proteins, including LptA-LptG [3], with lipoproteins LptM and LptY (formerly YedD) recently described as accessory components of the outer-membrane LptDE translocon [4]. LPS is transferred from the inner membrane by the ATP-binding cassette transporter LptB_2_FG to LptC, carried across the periplasm through the LptA-containing bridge, and inserted into the outer membrane by the LptDEMY. LptA forms head-to-tail oligomers by β-strand complementation and constitutes the central periplasmic segment of this bridge. Disruption of the Lpt complex blocks LPS transport, impairs outer-membrane biogenesis, and leads to cell death. Several structurally distinct inhibitors exploit different functional nodes of the Lpt pathway: (i) the β-hairpin peptidomimetic murepavadin/POL7080 targets the LptD-containing outer-membrane translocon [5]; (ii) zosurabalpin and related macrocyclic peptides inhibit the LptFG by trapping its LPS-bound conformation [6]; and (iii) small-molecule hits such as IMB-881 [7] and IMB-0042 [8] interfere with the LptA-LptC interaction. These examples identify multiple components of the Lpt machinery as potential targets for novel antibiotics against Gram-negative bacteria.

Another Lpt-targeting compound is thanatin, a disulfide-stabilized β-hairpin antimicrobial peptide isolated from the spined soldier bug *Podisus maculiventris* [9]. Thanatin exhibits potent activity against Gram-negative pathogens, including multidrug-resistant strains of *Escherichia coli* and *Klebsiella pneumoniae*. It is one of the first examples of natural peptides that inhibit Gram-negative bacteria by targeting the Lpt complex rather than acting through nonspecific membrane disruption. Thanatin has been reported to bind LptA and the outer-membrane LptD/E complex [10], but genetic resistance data point to LptA as the main target relevant to thanatin antibacterial activity. By binding LptA, thanatin disrupts formation of the LptA-centered periplasmic bridges, including LptA-LptA and LptA-LptC interactions.

Previous structure-activity relationship (SAR) studies have shown that thanatin activity depends on a constrained β-hairpin scaffold and several functionally important motifs. The C11-C18 disulfide bridge stabilizes the β-hairpin structure, whereas disruption of the disulfide bond, substantial *N*-terminal truncation, or changes in the loop geometry reduce its antibacterial activity [9,11]. Both the cationic loop, including R13 and R14 residues, and the *C*-terminal Gln-Arg-Met region contribute to potent activity against Gram-negative bacteria and Lpt engagement [12]. Consequently, recent LptA-focused optimization studies have shown that thanatin-derived peptidomimetics acted through stereospecific disruption of the Lpt periplasmic bridge, and modification of the *C*-terminal residue, including substitution with an aromatic one, could strongly influence antibacterial activity and target binding of optimized analogs [13]. These findings identified the *C*-terminal region as a key site for rational design of thanatin-like antibiotics.

Complementary SAR studies and analysis of natural libraries of thanatin-like peptides allow for the identification of sequence features associated with enhanced LptA-directed activity. Heteropteran thanatin-like peptides share a compact disulfide-stabilized β-hairpin fold but differ in primary structures, charge distribution, hydrophobicity, and *C*-terminal residue identity [14,15]. In our previous work, we have performed an expanded biological characterization of Rip-2, a thanatin-like peptide from *Riptortus pedestris*, and have shown that it was more active than thanatin against a panel of Gram-negative bacteria [15]. Rip-2 shares an LptA-directed mode of action with thanatin, but the molecular basis for its enhanced activity remained unclear. Here, we investigated the molecular features associated with the enhanced activity of Rip-2 compared to thanatin. We show that Rip-2 produces a concentration-dependent MST response with LptA_m_-sfGFP-His_6_, consistent with interaction under the assay conditions used; however, these data do not support a stronger LptA_m_-associated response as a simple explanation for its enhanced antibacterial activity. Using a phenotypic counter-screening, designed to reveal Rip-2-selective reduced susceptibility, we identified several rare mutants carrying the same LptA[R76C] substitution. The *C*-terminal mutagenesis of Rip-2, bioinformatic analysis of natural thanatin-like homologs, and structure-guided modeling support a scenario in which the *C*-terminal phenylalanine of Rip-2 may contribute to an LptA R76-dependent aromatic interaction. These findings open the door for an optimization of thanatin-like peptide structures and provide a framework for designing LptA-targeting peptide antibiotics.

## 2. Results and Discussion

### 2.1. Rip-2 Exhibits an Enhanced Antibacterial Activity Without Detectable Disruptions of Bacterial Membranes

**Figure 1:**
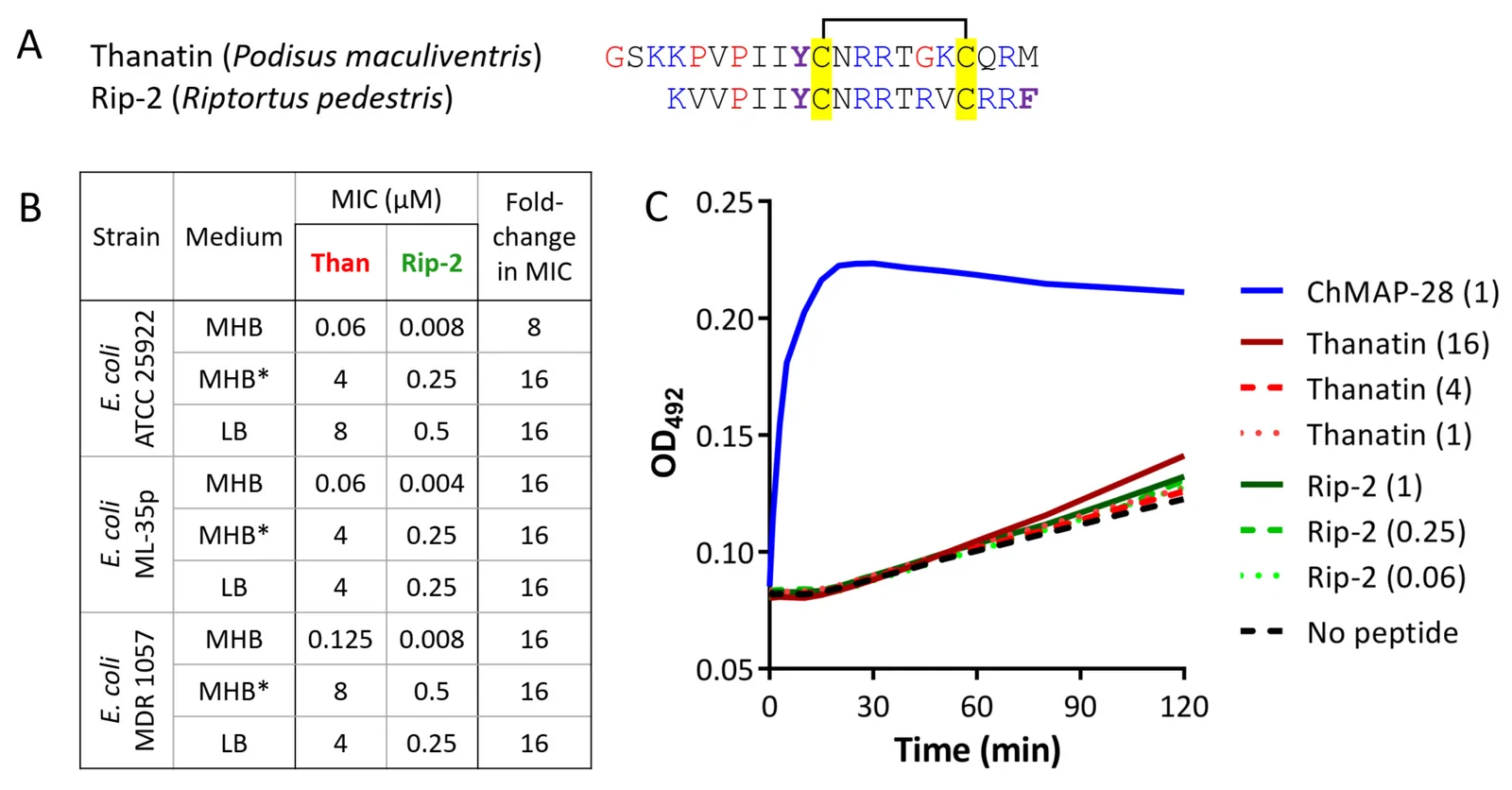
Antibacterial activity and effect on outer membrane permeability displayed by thanatin and Rip-2 in *E. coli*. (**A**) Amino acid sequence alignment of thanatin and Rip-2. The disulfide bond is marked with a square bracket. (**B**) Antibacterial activity of Rip-2 and thanatin (Than) against *E. coli* strains in different culture media. *—MHB supplemented with 0.9% NaCl. (**C**) Effect of Rip-2, thanatin, and the membrane-active control peptide ChMAP-28 on the outer membrane permeability of *E. coli* ML-35p, assessed by nitrocefin hydrolysis. Values in brackets indicate peptide concentrations in μM. Two independent experiments were performed, and similar curve patterns were observed.

In contrast to the structurally related membrane-active β-hairpin peptides Rip-3 and Rip-4, both Rip-2 and thanatin (Figure 1A) have earlier been shown to cause no detectable cytoplasmic membrane permeabilization in *E. coli* ML-35p [15]. However, the molecular basis for the enhanced activity of Rip-2 relative to thanatin remained unclear. To further compare Rip-2 and thanatin, we determined their minimum inhibitory concentration (MIC) values against three *E. coli* strains in different media: Mueller-Hinton broth (MHB), MHB supplemented with 0.9% NaCl, and lysogeny broth (LB) medium (Figure 1B).

Under all test conditions, Rip-2 was consistently more active than thanatin, with MIC values up to 16-fold lower. As Rip-2 has a higher GRAVY (grand average of hydropathy) index than thanatin (−0.23 versus −0.81), we decided to elucidate whether its increased hydrophobicity could enhance penetration of the Gram-negative bacterial outer membrane and thereby facilitate access to the periplasmic target LptA. To examine this possibility, outer membrane permeabilization of *E. coli* ML-35p was analyzed using a nitrocefin hydrolysis assay (Figure 1C).

In contrast to the membrane-disruptive peptide ChMAP-28, used as a positive control, neither Rip-2 nor thanatin induced a rapid increase in nitrocefin hydrolysis at MIC-level concentrations. The kinetic curves for both thanatin and Rip-2 remained close to that of the untreated control peptide, indicating that an enhanced activity of the latter is unlikely to be explained by strong nonspecific outer-membrane permeabilization under the conditions tested. At the same time, nitrocefin hydrolysis requires sufficient permeability for a relatively polar β-lactam substrate so that it could reach the periplasmic β-lactamase and may not capture more subtle changes in LPS packing, surface hydrophobicity, or local membrane organization. Previous studies have shown that thanatin could perturb the Gram-negative outer membrane through displacement of divalent cations and LPS release, and that thanatin-derived peptides could increase NPN fluorescence, consistent with increased access of this hydrophobic probe to the outer membrane [16]. Notably, a stronger NPN fluorescence for the *C*-terminal Phe-containing VF16 peptide than for related thanatin-derived analogs has been reported [17]. Thus, although Rip-2 and thanatin did not produce a ChMAP-28-like nitrocefin response, a limited LPS-level outer-membrane perturbation or an improved access to the periplasm cannot be excluded. Such effects could contribute to activities against Gram-negative bacteria, but our data obtained with nitrocefin argue against a strong nonspecific outer-membrane permeabilization as the most probable explanation for the enhanced activity of Rip-2.

### 2.2. Rip-2 Produces a LptA-Associated MST Response Distinct from That of Thanatin

**Figure 2:**
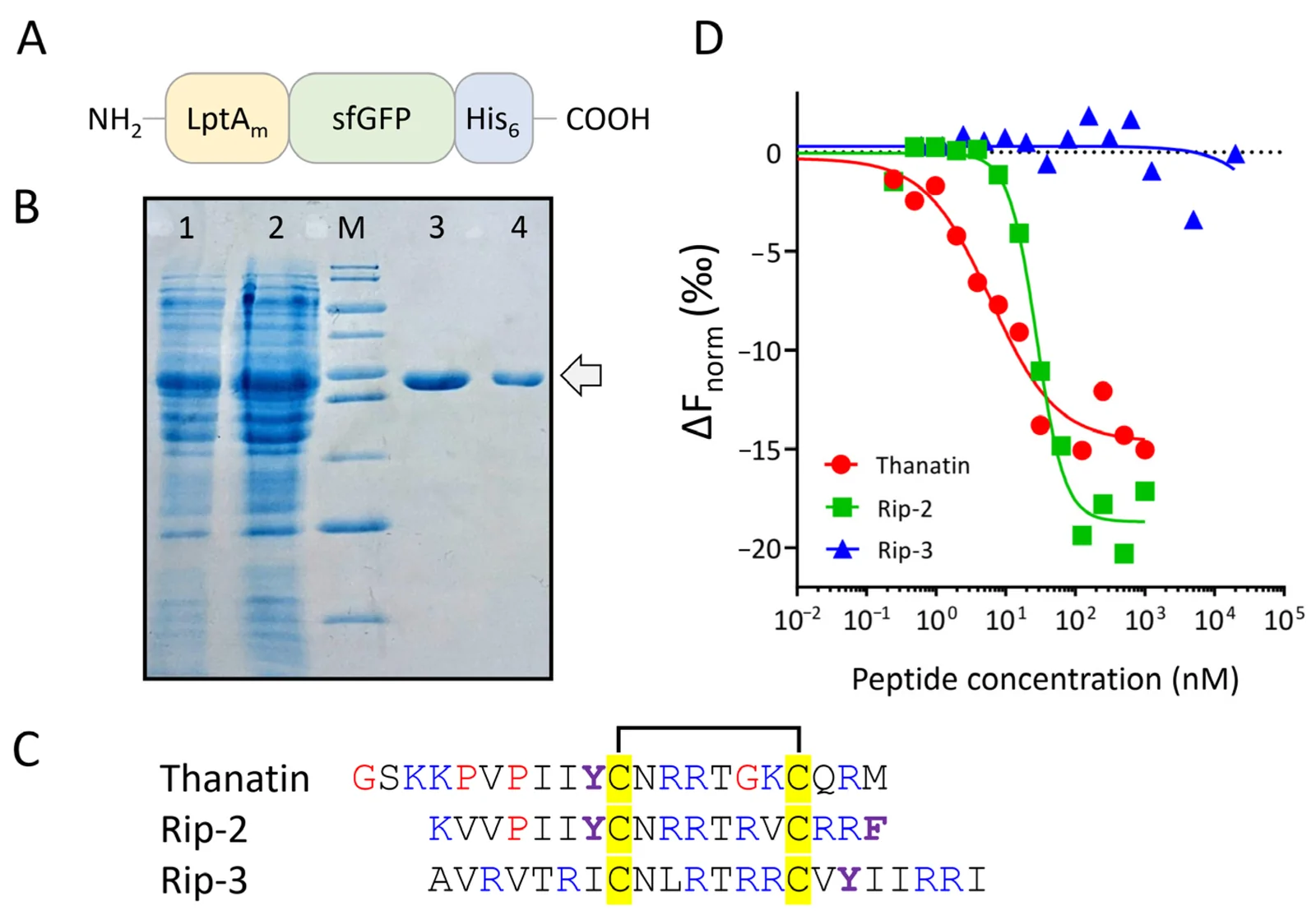
Microscale thermophoresis analysis of Rip-2 and thanatin interaction with LptA_m_-sfGFP-His_6_. (**A**) Schematic representation of the LptA_m_-sfGFP-His_6_ fusion protein. (**B**) SDS-PAGE analysis of the recombinant LptA_m_-sfGFP-His_6_ expression and purification. Lanes 1–2: whole-cell lysates before the IPTG induction, with the 2-fold sample amount loaded onto lane 2 as compared to lane 1; lanes 3–4: the recombinant protein after purification using IMAC, with the 2-fold sample amount loaded onto lane 3 as compared to lane 4. (**C**) Amino acid sequences of the peptides used in the MST assay. The disulfide bond is marked with a square bracket. (**D**) MST curves for the fluorescent LptA_m_-sfGFP-His_6_ titrated with unlabeled Rip-2 or thanatin. Rip-3 was used as a negative control. Measurements were performed using 300 nM target fusion protein, which was the lowest concentration that provided sufficient fluorescence intensity for reliable measurements with the fusion protein, at 40% MST power and 80% excitation power. Two independent experiments were performed (Figure S1, Table S1).

The lack of outer membrane-disruptive effects observed for Rip-2 and thanatin prompted us to compare their interactions with LptA, the periplasmic component of the LPS transport bridge targeted by thanatin-like peptides. Microscale thermophoresis (MST) was selected to analyze peptide-protein interactions in solution under near-physiological conditions. As the full-length LptA can self-associate through β-strand complementation, we used a monomeric truncated variant, LptA_m_, lacking the *C*-terminal β-strand. This construct has been previously used to reduce LptA aggregation while preserving the peptide-binding interface [10,18]. To avoid direct chemical modification of the relatively small peptide ligands, Rip-2 and thanatin were kept unlabeled. Instead, the fluorescence in the MST experiments was measured using LptA_m_ expressed as the *C*-terminal sfGFP fusion protein containing a His_6_ tag (Figure 2A). The recombinant LptA_m_-sfGFP-His_6_ was expressed in *E. coli* BL21(DE3) and purified by Ni-NTA affinity chromatography. SDS-PAGE analysis revealed a major band at the *M_r_* value of approximately 43 kDa, which was consistent with the calculated molecular mass of the fusion protein (Figure 2B).

In the MST assay, fluorescent LptA_m_-sfGFP-His_6_ was titrated with unlabeled peptides. Thanatin was included as a positive control, whereas Rip-3, a structurally related β-hairpin peptide acting against *E. coli* through membrane disruption rather than via a LptA-directed mechanism, was used as a negative control (Figure 2C). Previous work reported nanomolar-range binding of thanatin to DyLight650-labeled LptA_m_ [10]. In our sfGFP-based MST assay, both thanatin and Rip-2 exhibited clear concentration-dependent responses with LptA_m_-sfGFP-His_6_ (Figure 2D). In both independent experiments (Figure 2D, Figure S1, Table S1), Rip-2 showed a right-shifted response profile compared with thanatin. However, because the concentration of LptA_m_-sfGFP-His_6_ was 300 nM, and the fitted values varied between independent experiments, these parameters should be interpreted as apparent assay-dependent values rather than precise equilibrium dissociation constants. In contrast, Rip-3 did not produce a detectable saturable MST response at concentrations up to 20 µM, supporting the specificity of the observed effects. The finding that Rip-2 did not show a stronger apparent response profile for LptA_m_ than thanatin under the MST conditions used, despite its higher antibacterial activity, indicates that the enhanced potency of Rip-2 cannot be explained simply by stronger apparent target binding. A similar lack of direct correlation between target binding measurements and antimicrobial activities has been observed for thanatin analogs, where improved binding to LptA does not necessarily result in lower MIC values against *E. coli* [14]. Thus, the antibacterial potency of a thanatin-like peptide likely depends not only on apparent interaction with LptA, but also on its access to the periplasm and ability to disrupt interactions mediating the Lpt bridge assembly in vivo.

Although both thanatin and Rip-2 showed concentration-dependent MST responses with LptA_m_, the corresponding response profiles were not identical. Thanatin caused a more gradual concentration-dependent transition, whereas Rip-2 generated a steeper curve and a larger thermophoretic shift. As MST signal amplitude and curve shape can be influenced by changes in hydration shell, charge distribution, conformation, hydrodynamic properties, or oligomeric state of the complex, the observed differences should not be interpreted as evidence of stronger binding. Rather, they may reflect partially distinct LptA_m_-associated responses to Rip-2 and thanatin, or different changes upon complex formation. Together with the absence of major differences in membrane-permeabilizing activity, these data support a shared LptA-directed mode of action of both peptides but point to differences in molecular details of the target engagement.

### 2.3. A Phenotypic Counter-Screening Identifies Rare Mutants with Rip-2-Selective Resistance Phenotype

**Figure 3:**
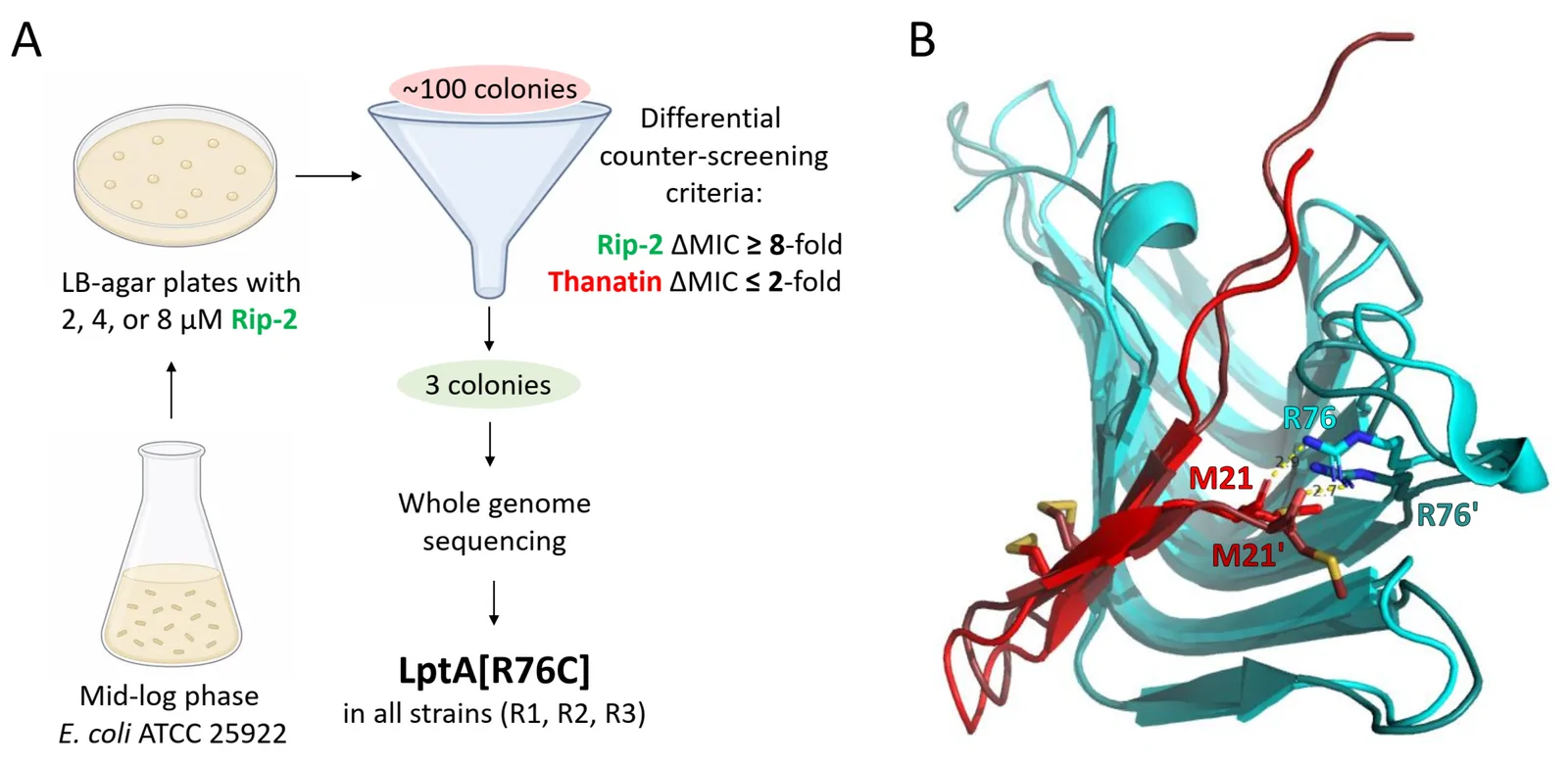
Differential phenotypic screening. (**A**) Schematic representation of the differential screening strategy used to identify clones with increased Rip-2-resistance but minimal cross-resistance to thanatin. The R76C mutant strains R1, R2, and R3 are specified in parentheses. (**B**) Overlay of the experimentally determined LptA_Eco_-thanatin complexes obtained by NMR (PDB ID: 6GD5, burgundy) and X-ray crystallography (PDB ID: 8GAJ, red). Interatomic distances are indicated in Å.

As the higher antibacterial activity of Rip-2 could not be explained simply by a stronger LptA-associated MST response, we considered it necessary to identify possible genetic changes associated with differential activities of Rip-2 and thanatin. In our previous experiments on the Rip-2-resistance selection, conventional serial passages recovered mutant strains carrying LptA[E84D] or LptA[D41G] phenotypes [15]. Since these mutants showed a pronounced cross-resistance to thanatin, they likely reflected common mechanisms of resistance development within the LptA-targeting thanatin-like peptide family rather than determinants specific to Rip-2. We therefore modified our selection strategy to enrich for clones with a selectively reduced susceptibility to Rip-2 while simultaneously retaining a near-wild-type susceptibility to thanatin (Figure 3A).

Accordingly, 1 × 10^8^ CFU of *E. coli* ATCC 25922 were plated on agar containing Rip-2 at concentrations of 2, 4, or 8 µM, corresponding to the MIC range of thanatin and exceeding the MIC of Rip-2 against the wild-type *E. coli* strain by 8-, 16-, or 32-fold. Resistant colonies were recovered at the peptide concentrations of 2 µM (~90 colonies), 4 µM (10 colonies), and 8 µM (3 colonies). All individual colonies were then screened on the basis of differential susceptibilities to Rip-2 and thanatin. Only clones showing at least an 8-fold increase in the Rip-2 MIC, but no more than a 2-fold increase in the thanatin MIC, were selected for further analysis. Among the screened colonies, only three clones met these criteria (Figure 3A). Thus, Rip-2-specific resistance was rare, suggesting that only a limited number of genetic changes could impair the Rip-2 antimicrobial activity without causing a broad cross-resistance to thanatin. Whole-genome sequencing of these three clones revealed that all selected clones carried the same single nucleotide polymorphism (SNP) in the *lptA* gene: C→T transition converting the R76 codon CGT to the cysteine codon TGT, resulting in the LptA[R76C] substitution. Notably, in two of the three selected clones, LptA R76C was the only detected gene difference relative to the parental strain (Table S2). The third clone, R3, also carried an additional *cpdA* Q19stop mutation; therefore, the phenotype of this clone cannot be attributed to R76C alone. R76C mutation was further confirmed by PCR amplification of *lptA* from the resistant clones followed by Sanger sequencing of the resulting amplicons.

In experimentally resolved LptA_Eco_-thanatin complex structures by NMR [10] or X-ray [14] techniques, the *C*-terminal M21 residue of thanatin is closely spaced to LptA[R76], and the R76 residue stabilizes the peptide *C*-terminus conformation through a bidentate salt bridge with its *C*-terminal carboxylate (Figure 3B). However, the preserved antimicrobial activity of the *C*-terminally amidated thanatin [9] indicates that this carboxyl group is not an absolute requirement for exhibition of the native thanatin activity. This is consistent with a distributed binding mode in which multiple contacts contribute to thanatin interaction with LptA. Therefore, the repeated recovery of the R76C substitution pointed to the *C*-terminal peptide-binding region of LptA as a likely factor affecting the functional difference between Rip-2 and thanatin, but also suggested that the effect of this amino acid substitution on Rip-2 activity cannot be explained only by loss of a canonical interaction between guanidinium and carboxyl groups. Instead, we hypothesized that the antimicrobial activity of Rip-2 was more dependent on its *C*-terminal F18 residue, which might interact with LptA through both the peptide *C*-terminal carboxyl group and its aromatic side chain. To test this hypothesis, we generated Rip-2 analogs with substitutions of the *C*-terminal phenylalanine and analyzed their activity against the wild-type *E. coli* and the LptA[R76C] mutant strains.

### 2.4. C-Terminal Phenylalanine May Serve as an Aromatic Anchor in the R76-Dependent Engagement of Rip-2

**Figure 4:**
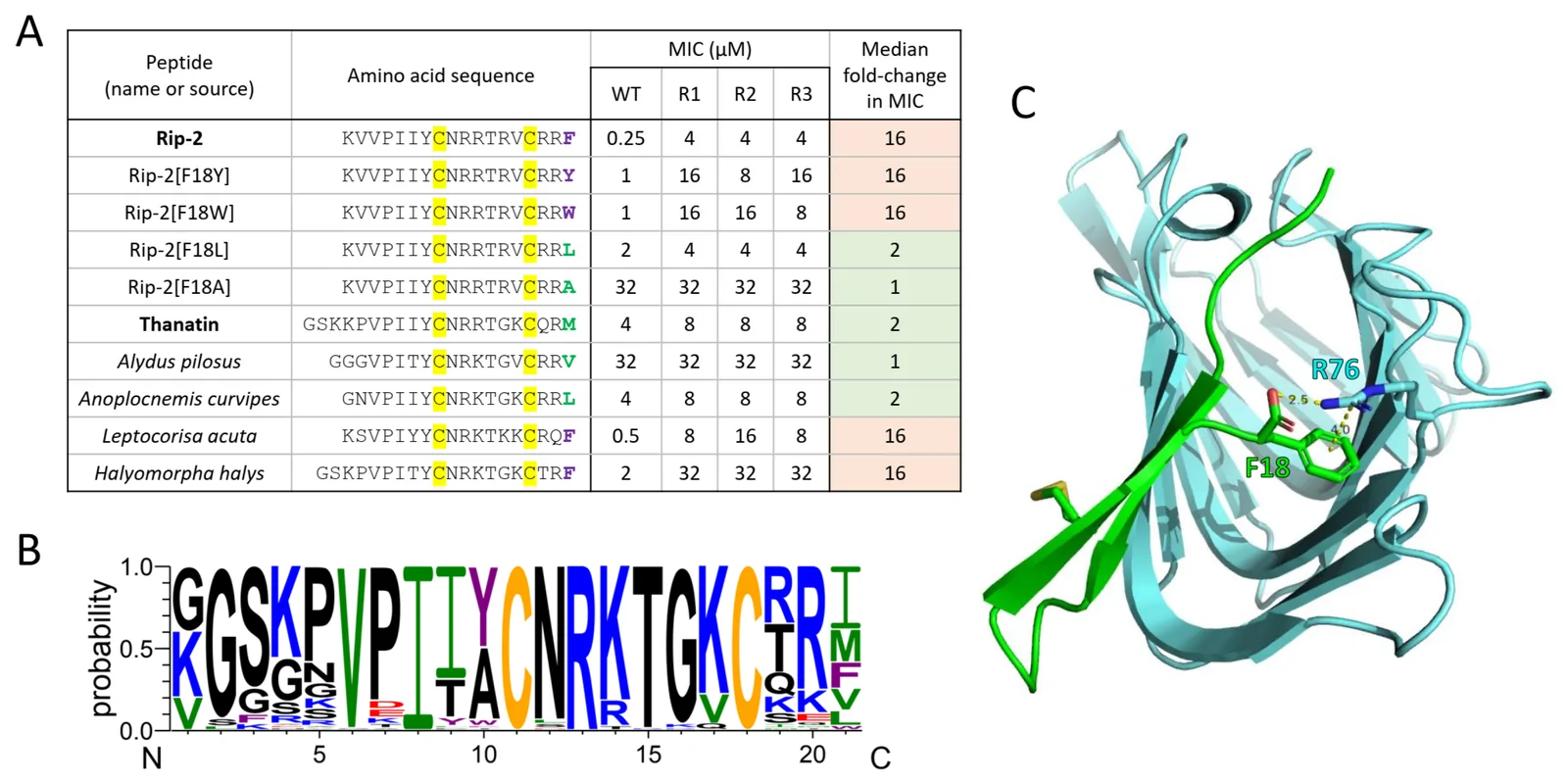
Structure-activity relationship analysis of thanatin-like peptides. (**A**) Antibacterial activities of thanatin-like peptides against the wild-type *E. coli* ATCC 25922 (WT) strain and three independently isolated LptA[R76C] mutant strains (R1, R2, R3). Aromatic *C*-terminal residues are indicated in purple, whereas aliphatic *C*-terminal residues are shown in green. Disulfide bridges are highlighted in yellow. (**B**) Sequence logo of thanatin-like peptides generated from a library of 75 natural homologs. (**C**) AlphaFold3 model of the LptA_Eco_-Rip-2 complex. LptA is shown in cyan and Rip-2 is colored in green. Interatomic distances are indicated in Å.

A panel of Rip-2 analogs, in which the *C*-terminal F18 residue was replaced with aromatic (Y or W) or aliphatic (A or L) residues, was generated and tested against the wild-type *E. coli* ATCC 25922 and the LptA[R76C]-containing mutant strains (Figure 4A). In parallel, we analyzed four natural thanatin-like homologs bioinformatically identified in genomes or transcriptomes of different heteropteran species (Figure 4A, Table S3). These peptides differed in their primary structures and carried distinct hydrophobic *C*-terminal amino acid residues. Unrelated AMPs (β-hairpin tachyplesin-1 from the Japanese horseshoe crab, human α-helical cathelicidin LL-37, and the bovine proline-rich peptide Bac7_1-35_) and polymyxin B were used as control compounds.

Substitution analysis showed that F18 is an important factor affecting the Rip-2 antimicrobial activity. The replacement F18A strongly impaired the activity against the *E. coli* ATCC 25922 strain (MIC of 32 µM), whereas the substitution F18L, which keeps the peptide hydrophobicity but loses an aromatic π-system, caused an approximately 8-fold increase in the MIC value. The unmodified Rip-2, which carries the *C*-terminal phenylalanine, was the most active peptide among all the Rip-2 analogs tested against *E. coli*, indicating that this residue is particularly important and favorable within this scaffold. However, a *C*-terminal aromatic residue alone is insufficient to enhance antimicrobial potency. The M21F substitution in thanatin did not reduce the MIC value against *E. coli*, despite a reported enhanced binding of this analog to LptA relative to thanatin [12], and conservative F18Y or F18W substitutions in Rip-2 reduced antimicrobial activities of these analogs by approximately 4-fold, with MIC values of 1 µM. Therefore, the functional contribution of a *C*-terminal aromatic residue depends on its structural context. Sequence-logo analysis of 75 bioinformatically identified thanatin-like peptides (Table S3) showed that the *C*-terminal position is restricted to hydrophobic residues, predominantly I, M, F, V, and L, with the more rarely occurring W (Figure 4B). This suggests a selective pressure to maintain a hydrophobic *C*-terminus, while the occurrence of F and W in a subset of homologs indicates that aromatic *C*-terminal residues have evolved in some thanatin-like peptides.

Analysis of Rip-2-resistant strains carrying the R76C substitution revealed a clear dependence of thanatin-like peptide activity on the *C*-terminal residue type (Figure 4A). Rip-2 analogs and natural thanatin-like homologs bearing aromatic *C*-terminal residues showed a pronounced loss of activity against the R76C-containing resistant strains (R1, R2, R3), with MIC value increases of up to 16-fold (Figure 4A). In contrast, the peptides with aliphatic *C*-terminal residues were only weakly affected or unaffected. Antimicrobial activities of control antimicrobial agents were not changed against all tested strains, with MIC values of 0.125, 0.25, 4, and 1 µM for polymyxin B, tachyplesin-1, LL-37, and Bac7_1-35_, respectively. Thus, within the tested panel, the R76C-containing strains did not show broad antimicrobial resistance but selectively compromised thanatin-like peptides bearing aromatic *C*-terminal residues.

To rationalize the selective effect of the R76C substitution, we used AlphaFold3 [19] to model the interaction between Rip-2 and LptA_m_ from *E. coli* ATCC 25922. In the predicted complex, the *C*-terminal region of Rip-2 is positioned near the R76 residue of LptA (Figure 4C). The R76 guanidinium group lies close (~2.5 Å) to the free *C*-terminal carboxyl group of Rip-2, which is consistent with a possible salt bridge formation or hydrogen-bonding interaction, and it is also localized closely (~4 Å) to the aromatic ring of the F18 residue of Rip-2, suggesting a possible cation-π interaction. Although experimental structural validation is required, the model provides a plausible structural explanation for the resistance-associated and antimicrobial activity (MIC) data. This model is consistent with that previously proposed for optimized thanatin-derived peptidomimetics targeting the Lpt transport bridge [13]. In particular, the optimized compound 7 comprises a *C*-terminal Tyr-containing scaffold [13]. In their LptA-bound model, the *C*-terminal carboxyl group forms a salt bridge with the R76 guanidinium group of LptA, and this interaction was proposed to be further stabilized by a cation-π contact between the R76 residue of LptA and the *C*-terminal Y21′ residue of the peptide [13]. This resembles the interaction proposed here for Rip-2, in which the R76 residue of LptA may engage the peptide *C*-terminal carboxyl group and may also interact with the aromatic side chain of the F18 residue. Thus, optimization of the thanatin structure by methods of medicinal chemistry and natural sequence variation in Rip-2 converge on a related local interaction principle: a *C*-terminal aromatic residue of AMP may strengthen the LptA engagement when it is properly positioned relative to the R76 residue of LptA. Our data extend this concept genetically by linking the R76C substitution to reduced activity of Rip-2 and other thanatin-like peptides bearing aromatic *C*-termini.

Together, these results allow us to propose a model in which the R76 residue of LptA may act as a bifunctional residue, potentially engaging both the Rip-2 *C*-terminal carboxyl group and aromatic side chain. Replacement of R76 with cysteine could disrupt this two-component anchoring module, providing a plausible explanation for the selective loss of the Rip-2 antimicrobial activity. Therefore, we propose that the enhanced activity of Rip-2 may involve an LptA R76-dependent contribution of its *C*-terminal aromatic residue, providing a structural principle that might be considered in the design of improved thanatin-like antibiotics.

## 3. Materials and Methods

### 3.1. Identification and Production of Thanatin-like Peptides

AMP genes were identified by TBLASTN searches against RNA-seq Sequence Read Archive (SRA) and GenBank whole-genome sequencing (WGS) datasets available for *Heteroptera* species, using precursor proteins of all known thanatin-like peptides [14,15] as queries. Searches were performed with default parameters, including the BLOSUM62 matrix and gap costs of 11 for gap opening and 1 for gap extension. The resulting hit sequences were manually inspected to identify putative mature AMP-coding regions, defined as sequences located between the predicted furin-like protease cleavage site and the stop codon (Table S3). Codon-optimized DNA fragments encoding the four selected peptides from *A. pilosus*, *A. curvipes*, *L. acuta*, and *H. halys* were generated by annealing partially self-complementary oligonucleotides followed by PCR amplification and cloned into a pET-based vector as described previously [15]. Rip-2 variants carrying substitutions at the *C*-terminal residue were generated by site-directed mutagenesis of the Rip-2 expression plasmid, whereas coding sequences for the selected thanatin-like homologs were assembled from overlapping oligonucleotides and cloned into the expression vector using the previously described procedure [20]. All recombinant peptides, including Rip-2, Rip-3, Bac7_1-35_ and ChMAP-28, the Rip-2 modified analogs, and the thanatin-like homologs, were expressed in *E. coli* BL21(DE3) as fusion proteins with a His-tag and the modified thioredoxin A carrier as previously described [15]. Briefly, transformed *E. coli* BL21 (DE3) cells were grown up at 37 °C to OD_600_ 1.0 in LB medium containing 20 mM glucose, 100 μg/mL of ampicillin, 1 mM MgSO_4_, 50 mM Na_2_HPO_4_, 50 mM KH_2_PO_4_, 25 mM (NH_4_)_2_SO_4_, and then were induced with 0.3 mM of isopropyl β-D-1-thiogalactopyranoside (IPTG). The induction was performed for 16 h at 30 °C, with a shaking speed of 220 rpm. Purification of the target peptides was performed by immobilized metal affinity chromatography (IMAC), BrCN cleavage of the fusion protein, and reverse-phase high-performance liquid chromatography (RP-HPLC). RP-HPLC peaks collected by monitoring at 214 and 280 nm were analyzed by MALDI-TOF mass spectrometry. The obtained fractions with confirmed molecular masses (Table S4) were dried and dissolved in water. Thanatin and LL-37 (of >98% purity for both peptides) were synthesized by Syneuro LLC (Moscow, Russia) using standard solid-phase methods.

### 3.2. Evaluation of Antibacterial Activity

Minimum inhibitory concentrations (MICs) of the peptides were determined by two-fold broth microdilution in 96-well microplates under three medium conditions: Mueller-Hinton broth (MHB), MHB supplemented with 0.9% NaCl, and LB medium. Single colonies of the tested *E. coli* strains were inoculated into 1 mL of the corresponding medium and grown at 37 °C to an OD_600_ of approximately 1.0. Cultures were diluted to 2 × 10^6^ CFU/mL in 2 × MHB, 2 × MHB supplemented with 1.8% NaCl, or 2 × LB. Two-fold serial dilutions of the peptides were prepared in 0.1% bovine serum albumin (BSA) to minimize nonspecific adsorption to the plate surface and mixed with an equal volume of the bacterial suspension. Plates were incubated for 24 h at 37 °C with shaking at 800 rpm. The MIC was defined as the lowest peptide concentration that prevented detectable bacterial growth, as assessed visually and by absorbance at 600 nm. The results were expressed as the median values determined based on at least three independent experiments performed in triplicate.

### 3.3. Outer Membrane Permeabilization Assay

The effects of the peptides on outer membrane permeability were evaluated in *E. coli* ML-35p using nitrocefin (Calbiochem, Darmstadt, Germany) as a chromogenic substrate for constitutively expressed periplasmic β-lactamase. *E. coli* ML-35p was grown in tryptic soy broth (TSB) for 16 h at 37 °C, then cells were washed three times with phosphate-buffered saline (PBS; pH 7.4) and adjusted to 2.5 × 10^8^ CFU/mL. Reaction mixtures were prepared in 96-well plates in a final volume of 200 μL and contained 2.5 × 10^7^ CFU/mL cells and 25 μM nitrocefin. Plates were incubated at 35 °C with shaking at 500 rpm, and nitrocefin hydrolysis was monitored by recording absorbance at 492 nm using an AF2200 microplate reader (Eppendorf, Hamburg, Germany). Cells incubated with nitrocefin in the absence of peptide served as the negative control, whereas the membrane-active AMP ChMAP-28 was used as the positive control. Two independent experiments were performed, and similar curve patterns were observed.

### 3.4. Construction, Expression and Purification of the Recombinant LptA_m_-sfGFP-His_6_

Because full-length LptA tends to oligomerize in solution, we used LptA_m_, a truncated variant lacking the *C*-terminal β-strand that contributes to LptA self-association [10,18]. To enable fluorescence detection during microscale thermophoresis (MST) measurements, the plasmid encoding the LptA_m_-sfGFP-His_6_ fusion protein was assembled by ligation-independent cloning using PCR fragments with complementary ends. The LptA_m_ coding sequence was amplified from *E. coli* ATCC 25922 genomic DNA. The vector backbone, retaining the regulatory elements and the *sfgfp-his_6_* sequence but lacking *lptE*, was amplified from the previously described pHybr-LptE-sfGFP-His_6_ plasmid [21]. The vector amplicon was treated with DpnI to remove the methylated plasmid template. Purified insert and vector fragments were mixed at a 1:1 mass ratio and used to transform competent *E. coli* DH10B cells. Transformants were screened by colony PCR, and the correct assembly of the resulting plasmid was confirmed by Sanger sequencing. *E. coli* BL21(DE3) was transformed with the obtained plasmid, and a single colony was used to inoculate 10 mL of LB medium containing 20 mM glucose and 20 μg/mL chloramphenicol. The overnight culture was transferred into 200 mL of LB medium supplemented with 20 mM glucose, 20 μg/mL chloramphenicol, 1 mM MgSO_4_, 50 mM Na_2_HPO_4_, 50 mM KH_2_PO_4_, and 25 mM (NH_4_)_2_SO_4_. Cells were grown at 37 °C with shaking at 220 rpm until the optical density at 600 nm reached 1.0. Protein expression was induced by adding 0.2 mM IPTG, and the culture was incubated at 30 °C for 16 h. Cells were harvested by centrifugation, resuspended in 100 mM phosphate buffer (pH 7.8) containing 500 mM NaCl, 30 mM imidazole, 1 mM PMSF, and sonicated on ice. The clarified lysate was loaded onto Ni-NTA Sepharose equilibrated with the buffer. The fusion protein was eluted with the same buffer supplemented with 0.5 M imidazole. The eluate was desalted and exchanged into PBS (pH 7.4) using a PD-10 column (Cytiva, Marlborough, MA, USA).

### 3.5. SDS-PAGE Analysis of LptA_m_-sfGFP-His_6_

Aliquots of *E. coli* BL21(DE3) cultures expressing recombinant LptA_m_-sfGFP-His_6_ were collected by centrifugation and the obtained cell pellets were resuspended in denaturing sample buffer containing 100 mM Tris-HCl, 1% SDS, 8 M urea, 1% β-mercaptoethanol, and 0.01% Coomassie Brilliant Blue G-250, pH 6.6. Samples were heated at 99 °C for 10 min and resolved by SDS-PAGE using 15% resolving and 5% stacking gels. Electrophoresis was performed in Tris-glycine running buffer containing 25 mM Tris-HCl, 192 mM glycine and 0.1% SDS, pH 8.3, at a constant current of 12 mA for 90 min. Gels were fixed in 50% methanol and 10% acetic acid for 30 min and stained with 0.025% Coomassie Brilliant Blue G-250 in 10% acetic acid for 1 h. Excess stain was removed by incubation in 10% acetic acid for 20 h, after which gels were rinsed with water and imaged.

### 3.6. Microscale Thermophoresis (MST)

Binding of thanatin and Rip-2 to recombinant LptA_m_-sfGFP-His_6_ was assessed by MST using a Monolith NT.115 instrument (NanoTemper Technologies, Munich, Germany). Rip-3, a membrane-active thanatin homolog, was included as a negative control. LptA_m_-sfGFP-His_6_ was used at a final concentration of 300 nM, which was the lowest concentration that provided sufficient fluorescence intensity for reliable measurements with the fusion protein. The peptides were tested over a final concentration range of 1 nM to 1 mM. Serial peptide dilutions were prepared in an aqueous solution containing 0.1% BSA and mixed in equal volumes with the protein solution. BSA was required to maintain LptA_m_-GFP-His_6_ in a non-aggregated state under the assay conditions. The samples were centrifuged for 5 min at 10,000× *g* to remove any aggregates and loaded into Monolith capillaries (cat. no. MO-K022). Measurements were performed at 40% MST power and 80% LED power. Non-linear regression curves were generated using GraphPad Prism v.8.0.1 (GraphPad Software Inc., San Diego, CA, USA). Two independent experiments were performed, and similar curve patterns were observed.

### 3.7. Selection of E. coli Clones with Reduced Susceptibility to Rip-2

Aliquots of an *E. coli* ATCC 25922 suspension containing 1 × 10^8^ CFU/mL were spread onto LB agar plates supplemented with Rip-2 at concentrations of 2, 4, or 8 μM. After overnight incubation at 37 °C, individual colonies grown on Rip-2-containing plates were transferred to LB medium and cultured to an OD_600_ of approximately 1.0. The MICs of Rip-2 and thanatin against each recovered isolate were determined in LB medium by two-fold broth microdilution as described above and compared with the corresponding MICs for the parental *E. coli* ATCC 25922 strain. Clones showing at least an 8-fold increase in Rip-2 MIC and no more than a 2-fold increase in thanatin MIC were selected for further analysis.

### 3.8. Whole-Genome Sequencing

Whole-genome sequencing of the selected resistant *E. coli ATCC* 25922 isolates was performed using the Illumina MiSeq platform (Illumina Inc., San Diego, CA, USA) with 2 × 100 bp paired-end reads using the previously described procedure [20]. Briefly, read quality was assessed with FastQC v0.11.9, followed by trimming with Trimmomatic PE v0.39, and assembled using SPAdes v3.13.0. For variant identification, reads were aligned to the *E. coli ATCC* 25922 reference genome using BWA-MEM v0.7.17-r1188, and variants were called with VarScan v2.4.0 using a minimum variant allele frequency of 0.90. To prove the obtained whole-genome sequencing data, the *lptA* gene was amplified by PCR using specific primers and inserted into the pAL-2T vector (Evrogen, Moscow, Russia). The ligation products were transformed into chemically competent *E. coli* DH10B cells. The obtained plasmids were sequenced on both strands using the ABI PRISM 3100-Avant automatic sequencer (Applied Biosystems, Waltham, MA, USA).

## 4. Conclusions

In this study, we show that the enhanced antibacterial activity of Rip-2 relative to thanatin is not explained by a stronger apparent LptA-associated MST response, or by increased nonspecific disruption of the bacterial outer membrane at MIC-level concentrations. Although subtle differences in access to the periplasm cannot be fully excluded, our results point to possible determinants of Rip-2 potency at the level of productive LptA engagement. A phenotypic counter-screening allowed us to reveal the Rip-2-selective resistance and identify the same LptA[R76C] substitution in independent resistant clones. This substitution was associated with only a limited effect on thanatin but strongly impaired the Rip-2 antibacterial activity, implicating the R76 residue of LptA as one that contributes to the differential interactions with these closely related thanatin-like peptides and, as a consequence, to their differing antibacterial activities.

*C*-Terminal mutagenesis of Rip-2, analysis of natural thanatin-like homologs, and structure-guided modeling allow us to propose a testable model in which the *C*-terminal phenylalanine of Rip-2 may contribute to an R76-dependent mode of target engagement. In this model, LptA R76 may help stabilize the peptide *C*-terminus and favor contact with the aromatic side chain of the terminal residue. A stronger effect of the R76C substitution on peptides with aromatic *C*-termini, together with a weaker effect on peptides bearing aliphatic *C*-terminal residues, suggests that natural thanatin-like peptides can use distinct local interaction modules while retaining the same general LptA-directed mode of action. Although the current data point at a functional link between the Arg76 residue of LptA and the *C*-terminal Phe18 residue of Rip-2, a direct validation using engineered R76C bacterial strains, quantitative binding measurements with the wild-type and mutant LptA_m_ proteins in more native environments, as well as structural analysis will be required to further validate this model and to define whether the proposed aromatic anchor operates within the full Lpt transport bridge.

Our results also show that natural sequence variation can help to identify amino acid residues and local interaction motifs that influence antimicrobial activities of AMPs. Together with resistance genetics and targeted mutagenesis, analysis of natural peptide variation can link specific sequence features of AMPs to measurable changes in their antibacterial activities. For thanatin-like peptides, the proposed R76-dependent contribution of the peptide *C*-terminal aromatic residue highlights a target-directed element that should be considered in future optimization of thanatin-like peptide structures. Such data may help to guide the design of improved LptA-targeting AMPs with increased activity against multidrug-resistant Gram-negative bacteria.

## Data Availability

Data on bacterial genome sequencing were deposited in the NCBI SRA database under the accession code PRJNA1514011. Additional data that support the findings of this study are available from the corresponding author upon reasonable request.

## Supplementary Materials

The following supporting information can be downloaded at: https://www.mdpi.com/article/10.3390/ijms27188072/s1.
