# Supplementary material for: The C-Terminal Phenylalanine of the Thanatin-like Peptide Rip-2 Contributes to Its Enhanced LptA-Associated Antimicrobial Activity Compared with Thanatin
Source: Int J Mol Sci. 2026 Sep 10;27(18):8072. doi: 10.3390/ijms27188072 (PMC13607708; doi:10.3390/ijms27188072)
Supplement: Supplementary file 1 [file ijms-27-08072-s001.zip › ijms-4482062-supplementary.pdf]

Supplementary Materials

The *C*-terminal phenylalanine of the thanatin-like peptide Rip-2 contributes to its enhanced LptA-associated antimicrobial activity compared with thanatin

Pavel V. Panteleev[1,2], Victoria N. Safronova[1], Yulia S. Teplovodskaya[1,2], Ilia Y. Toropygin[3], Ekaterina V. Nikitinskaya[4], Denis A. Nikitinskiy[4], Ilia A. Bolosov[1], Alexey V. Mishin[2], Tatiana V. Ovchinnikova[1,2,*]

[1] M.M. Shemyakin & Yu.A. Ovchinnikov Institute of Bioorganic Chemistry, Russian Academy of Sciences, Moscow, Russia; p.v.panteleev@gmail.com (P.V.P.); victoria.saf@ibch.ru (V.N.S.); juliyateplovodskaya@gmail.com (Y.S.T.); bolosov@ibch.ru (I.A.B.); ovch@ibch.ru (T.V.O.);
[2] Moscow Center for Advanced Studies, Moscow, Russia; mishinalexej@gmail.com (A.V.M.);
[3] Institute of Biomedical Chemistry, Moscow, Russia; toropygin@rambler.ru (I.Y.T.);
[4] All-Russian Plant Quarantine Center (VNIIKR), Bykovo, Ramensky district, Moscow Region, Russia; nikitinskajacat@yandex.ru (E.V.N.); denpreffect@gmail.com (D.A.N.).

* Correspondence: ovch@ibch.ru

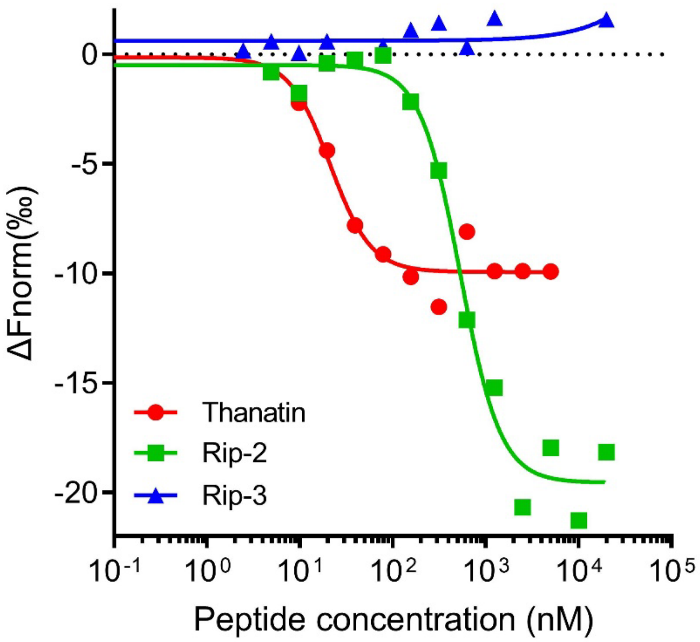

Figure S1. Independent replicate of MST binding analysis of Rip-2 and thanatin with LptA$_m$-sfGFP-His$_6$ (Experiment 2). Rip-3 was used as a negative control. Measurements were performed using 300 nM target fusion protein at 40% MST power and 80% excitation power.

Table S1. Nonlinear regression parameters for MST response curves

Parameter*	Thanatin		Rip-2	
	Experiment 1	Experiment 2	Experiment 1	Experiment 2
**IC_{50}**	6.1 nM	21.07 nM	27.73 nM	531.4 nM
**Std. error**	1.64 nM	4.16 nM	2.51 nM	61.91 nM
**95% CI**	2.44 ÷ 9.76 nM	11.23 ÷ 30.92 nM	22.2 ÷ 33.27 nM	393.5 ÷ 669.4 nM
**Hill slope**	-0.915	-1.968	-2.166	-2.001
**R^2**	0.9693	0.9531	0.9885	0.9838
**Sy.x**	1.17	0.9594	1.026	1.264

* The values are reported as apparent fit parameters obtained by nonlinear regression in GraphPad Prism. As the MST assay was performed using 300 nM fluorescent $LptA_m$-sfGFP-His_6, these values should not be interpreted as precise equilibrium dissociation constants.

Table S2. Mutations identified in *E. coli* ATCC 25922 strains with stable induced resistance to Rip-2

Strain	BioSample N	SRA accession N	Sequencing depth (x)	Mean mapped coverage (×)	Mutation	Gene	Type	Gene product	Variant frequency (%)	Read depth
R1	SAMN62469202	SRX34830603	124	88.69	R76C (CGT→TGT)	*lptA*	single nucleotide polymorphism	LptA transporter	99.0	96
R2	SAMN62469203	SRX34830604	161	121.29	R76C (CGT→TGT)	*lptA*	single nucleotide polymorphism	LptA transporter	100	135
R3	SAMN62469204	SRX34830605	117	72.15	R76C (CGT→TGT)	*lptA*	single nucleotide polymorphism	LptA transporter	100	78
					Q19stop (CAA→TAA)	*cpdA*	nonsense	cAMP phosphodi esterase	100	44

Table S3. A database of thanatin-like peptides identified in genomes and transcriptomes of *Heteroptera* species

Origin	Sequence	Source data	Accession numbers
Aelia acuminata	GSKPVPIIYCNRRTGKCTRM	WGS, RNAseq	CAJVQU000000000.2; ERX9660713
	GSKPVPIIYCNRKTGKCTRM		
Aenictopecheidae sp.	APVDITYCNRRTGVCKKV	RNAseq	SRX2454792
Anasa tristis	GNVPIYYCNRKTGKCRRL	RNAseq	SRX2920096
	GNVPIYYCNRKTGKCRRF		SRX2920096
Anoplocnemis curvipes	GNVPIIYCNRKTGKCRRL	RNAseq	SRX247058
	GNVPITYCNRKTGKCSKF		
Anoplocnemis dallasi	GNVPITYCNRKTGKCRTF	RNAseq	SRX8474032
Anoplogonius nigricollis	SPVDIIACNRKTGKCQKI	RNAseq	SRX893562
	VLFRSVDIIACNRKTGKCQKI		
	SPGDIIACNRKTGKCQKI		
Apolygus lucorum	GGSVPITYCNRKTGVCRRV	RNAseq	SRX4156107
	GGAVPITYCNRKTGVCRRV		SRX4156107
	GGRVPIPYCNRKTGVCRRV		SRX4156107
Alydus pilosus	GGGVPITYCNRKTGVCRRV	RNAseq	SRX893489
Arma chinensis	GSKPVPIIFCNRRTGKCQRM	RNAseq	SRX6830878
Bagrada hilaris	GSKPVPIIACNRKTGKCRRI	RNAseq	SRX15957777-91
Boisea trivittata	GGKVKIIACNRTTGQCREI	RNAseq	SRX893495
	GGKVKIIACNRKTGQCREI		
Chinavia impicticornis	GSKPVPIIYCNRRTGKCTRM	RNAseq	SRX9233434
	GSKPVPIIACNRKTGKCTR		
	GSKPVPIIACNRKTGKCTRI		
Chinavia ubica	GSKPVPIIACNRKTGKCTRI	RNAseq	SRX535270
Cletus punctiger	GNVPIIYCNRKTGKCRRF	RNAseq	SRX8180625; SRX8201725; SRX8230641
	ENVPIIYCNRKTGKCRRF		
	GNVPIIYCNRKTGKCRRI		
Coridius chinensis	SPVEIIYCNRKTGKCQKV	RNAseq	SRX9155370
Dichelops melacanthus	GSNPVPIIACNRKTGKCTRI	RNAseq	SRX533488
	GSKPVTITACNRKTGKCTKI		
	GSKPVPIIACNRKTGKCTRI		
Dolycoris baccarum	GSKPVPIIYCNRKTGKCTRM	RNAseq	SRX8176834
Eocanthecona furcellata	GSKPVPIIACNRKTGVCRRI	RNAseq	SRX20940516
Erthesina fullo	GSKPVPIIACNRRTGKCSRI	RNAseq	SRX731614
	GSRPVPIIACNRRTGKCTRI		
	GSKPVPIIACNRRTGKCRRI		
Euschistus heros	GSKPVPITACNRKTGKCMRI	RNAseq	SRX4632325
	GSKPVPITACNRKTGKCTKI		SRX5974347
	GSKPVTITACNRKTGKCTKM		
Eysarcoris guttigerus	GSKPVPIIYCNRKTGKCTRL	RNAseq	SRX8180630
	GSKPVPIIYCNRKTGKCSRF		
	GSKPVPIIACNRKTGKCTRI		
Gonocerus acuteangulatus	RSVPIIYCNRRTGKCRRL	WGS	CAMPFC000000000.1
Halyomorpha halys	GSKPVPITYCNRKTGKCTRF	WGS, RNAseq	JMPT00000000.3; SRX20468988
Homoeocerus walkeri	GNVPIIYCNRKTGKCRRL	RNAseq	SRX2363516
Hygia opaca	GNVPITYCNRKTGKCRRV	RNAseq	SRX8474033

Leptocorisa acuta	KSVPIYYCNRKTKKCRQF	RNAseq	SRX8230637
	KSVPIYYCNRRTKKCRQF		
Murgantia histrionica	GSKPVPIIACNRKTGKCSRI	RNAseq	SRX1431662-4
	GSKPVPIIACNRKTGKCTRI		
	GSKPVPIIACNRKTGKCRRI		
Nezara viridula	GSKPVPIIACNRKTGKCTRI	WGS, RNAseq	CAKMRH000000000.1; SRX8230642
	GSKPVPILACNRKTGKCTR		
Orius niger	GSKRVPIIYCNRKTGKCTRM	RNAseq	SRX8298710
Picromerus lewisi	GSKPVPIIACNRKTGVCSRI	RNAseq	SRX6863076
Piezodorus guildinii	GSKPVPIIACNRKTGKCRRI	WGS, RNAseq	JAJSOM000000000.1; SRX12686285
Piezosternum calidum	SPVEITYCNRKTGKCKKV	RNAseq	SRX893510
Plautia crossota	KGFKGVPIIACNRKTGKCIMF	RNAseq	DRX043869
Plautia stali	KGFKGVPIIACNRKTGKCIRF	RNAseq	DRX111574
Podisus maculiventris	GSKKPVPIIYCNRRTGKCQRM	RNAseq	SRX3026795
	GSKKPVAIIYCNRRRGKCQRM		
Rhaphigaster nebulosa	RGVPIIWCDRKTGKCQRL	WGS	CANUHP000000000.1
	GVPIIWCSRKTGKCKRM		
	GVPITWCSRKTGKCKRM		
Riptortus pedestris	GRVPIIYCNRKTGVCKRM	WGS, RNAseq	SRX17809475
	GKVPIIYCNRKTGVCKRM		
	GKVPIIYCNRKTGVCKEV		
	KVVPIIYCNRRTRVCRRF		
Scotinophara lurida	GSKPVPIIYCNRKTGKCTRM	RNAseq	SRX8176836
Scutelleridae sp.	SPVEITYCLRKTGVCRRW	RNAseq	SRX893561
	SPVEITYCLRKTGVCKKW		
Stiretrus anchorago	GSKPVPIIACNRKTGKCTRI	WGS	WUAS00000000.2
Thyanta pallidovirens	GSKPVPITACNRKTGKCTRI	RNAseq	SRX15285601
Zicrona caerulea	GSKPVPIIACNRKTGKCQRM	RNAseq	SRX8218988
	GSKPVPIITCNRKTGKCQRI		
	GSKPVPIIACNRKTGKCQRL		

Table S4. Amino acid sequences and molecular masses of the peptides used in this study

Peptide	Origin	Sequence	Molecular mass, Da	
			Calculated $[M+H]^+$ value[1]	Measured value[2]
Rip-2	Rec	KVVPIIYCNRRTRVCRRF	2277.28	2277.14
Rip-2[F18Y]	Rec	KVVPIIYCNRRTRVCRRY	2293.27	2293.31
Rip-2[F18W]	Rec	KVVPIIYCNRRTRVCRRW	2316.29	2316.30
Rip-2[F18L]	Rec	KVVPIIYCNRRTRVCRRL	2243.29	2243.31
Rip-2[F18A]	Rec	KVVPIIYCNRRTRVCRRA	2201.25	2201.23
A. pilosus	Rec	GGGVPITYCNRKTGVCRRV	2034.06	2033.45
A. curvipes	Rec	GNVPIIYCNRKTGKCRRL	2089.14	2089.07
L. acuta	Rec	KSVPIYYCNRKTKKCRQF	2260.19	2260.08
H. halys	Rec	GSKPVPITYCNRKTGKCTRF	2254.17	2254.15
Rip-3	Rec	AVRVTRICNLRTRRCVYIIRRI	2728.60	2728.16
Rip-4	Rec	AARVTIIRIRNKRTGKVTIIVIRRK	2931.89	2931.24
ChMAP-28	Rec	GRFKRFRKKLKRLWHKVGPFVGPILHY	3364.00	3364.22
$Bac7_{1-35}$	Rec	RRIRPRPPRLPRPRPRPLPFPRPGPRPIPRPLPFP	4205.55	4205.24
Thanatin	Synt	GSKKPVPIIYCNRRTGKCQRM	2433.29	2433.35
LL-37	Synt	LLGDFFRKSKEKIGKEFKRIVQRIKDFLRNLVPRTES	4491.58	4491.40

[1] according to Expasy Compute pI/Mw tool

[2] monoisotopic m/z were measured using MALDI-TOF MS (the measured m/z values match well the corresponding calculated molecular masses)
